# Proteomics profiling of the honeybee parasite *Tropilaelaps mercedesae* across post-embryonic development

**DOI:** 10.1038/s41597-024-03355-4

**Published:** 2024-05-15

**Authors:** Qiaohong Wei, Jiangli Wu, Fengying Liu, Jiajing Sun, Weipeng Kang, Meijiao Zhao, Feng Wang, Chenhuan Zhang, Shufa Xu, Bin Han

**Affiliations:** 1 State Key Laboratory of Resource Insects, Institute of Apicultural Research, Chinese Academy of Agricultural Sciences, Beijing, 100193 China; 2 Institute of Horticultural Research, Shanxi Academy of Agricultural Sciences, Shanxi Agricultural University, Taiyuan, 030031 China; 3 Zhejiang Provincial Key Laboratory of Biometrology and Inspection & Quarantine, College of Life Sciences, China Jiliang University, Hangzhou, 310018 China

**Keywords:** Proteomics, Developmental biology

## Abstract

*Tropilaelaps mercedesae*, an ectoparasitic mite of honeybees, is currently a severe health risk to *Apis mellifera* colonies in Asia and a potential threat to the global apiculture industry. However, our understanding of the physiological and developmental regulation of this pest remains significantly insufficient. Using ultra-high resolution mass spectrometry, we provide the first comprehensive proteomic profile of *T. mercedesae* spanning its entire post-embryonic ontogeny, including protonymphs, deutonymphs, mature adults, and reproductive mites. Consequently, a total of 4,422 *T. mercedesae* proteins were identified, of which 2,189 proteins were significantly differentially expressed (FDR < 0.05) throughout development and maturation. Our proteomic data provide an important resource for understanding the biology of *T. mercedesae*, and will contribute to further research and effective control of this devastating honeybee pest.

## Background & Summary

The honeybee (*Apis mellifera*) is an important economic insect that is extensively reared around the world. The pollination services provided by honeybees make them irreplaceable in protecting biodiversity and maintaining food security^1,2^. Nevertheless, a variety of biotic and abiotic factors have put severe pressure on the health of honeybees, leading to increased attention on their survival^3,4^. Among the stresses, ectoparasitic mites are the most prominent biotic stressors threatening honeybees, and the *Varroa destructor* (Mesostigmata: Varroidae) has been a global epidemic^5–7^, making it one of the primary research focuses^8–10^. However, another obligate ectoparasitic mite, *Tropilaelaps mercedesae* (Mesostigmata: Laelapidae), which is widely prevalent in Asia and causes serious damage to *A. mellifera*, also poses a risk of global spread^11^.

*T. mercedesae* has shifted from its original host, the giant Asian honeybee species (*Apis breviligula*, *Apis dorsata*, and *Apis laboriosa*), to *A. mellifera* for over half a century, and has now well adapted to parasitizing its new host, feeding on it, and transferring viruses^12,13^. Similar to the *Varroa* mites, the life cycle of *T. mercedesae* also includes alternates between reproductive and dispersal stages. The entire reproductive stage unfolds within the capped brood cells, initiating with a mature female entering the cell just before capping and beginning to lay eggs about 10 hours after capping, laying eggs approximately every 24 hours. Upon hatching, the eggs develop to maturity after passing through the protonymph stage and the deutonymph stage. During this period, the foundress mite and her offspring feed on the hemolymph of honeybee pupae^14^. By the time their host bees emerge from the cell, almost all female *T. mercedesae* offspring have become mature adults, emerge together with their hosts, and enter the next reproductive cycle^15^. Unlike *Varroa* mites, the dispersal phase of *T. mercedesae* is much shorter and they do not feed on adults. Instead, they feed on young larvae in uncapped cells^16,17^, as their body size and mouthparts are not suitable for parasitizing and feeding on adult bees^18^. The combination of an accelerated reproductive rate, truncated life cycle, and abbreviated dispersal phase collectively underpin the rapid proliferation of *T. mercedesae* within honeybee colonies, potentially endowing it with a heightened capacity to compromise bee health compared to *Varroa*. However, current research on the molecular biology of *T. mercedesae* is still limited, constraining a profound understanding and effective control of it.

In the current study, applying ultra-high-resolution mass spectrometry, we performed the first comprehensive proteomic profiling of *T. mercedesae* across all post-embryonic developmental stages (i.e., protonymph, deutonymph, adult, and reproductive stages). The data generated will provide an important resource for understanding the biology and developmental regulation of *T. mercedesae*, and will help to develop effective treatments for this devastating honeybee pest.

## Methods

### Sample collection

*Tropilaelaps mercedesae* samples were collected from 12 honeybee (*Apis mellifera*) colonies in three apiaries (four colonies from each apiary) located in Beijing, Tianjin, and Hebei, China. All colonies were queen right and managed using standard apicultural practices, except they were not treated to control mite populations.

All *T. mercedesae* samples were collected from capped brood cells using a soft paintbrush and soft tweezers. Reproductive mites (Rep) were collected from brood cells containing white-eyed pupae. Protonymphs (Pro) and one deutonymphs (Deu) were sampled from brood cells containing purple-eyed pupae. Adult *T. mercedesae* (Adu) were collected from cells close to emerging (about one day before adult bees emerged).

For each group (protonymphs, deutonymphs, adults, and reproductive mites), collected mites from all 12 colonies were randomly allocated to one of three replicates (50 mg for each replicate). All samples were rinsed with PBS and air-dried to remove many contaminates that may have been attached to the cuticle, flash-frozen using liquid nitrogen, and stored at −80 °C for further processing.

### Preparation of peptide extracts for proteomic analysis

Mite samples were homogenized at 4 °C using SDT buffer (4% SDS, 100 mM Tris-HCl, 1 mM DTT, pH 7.6) for sample lysis and protein extraction. The contrition of protein was quantified with the BCA Protein Assay Kit (Bio-Rad, USA). The general quality of extracted proteins was confirmed by SDS-PAGE: 20 µg of protein for each sample were mixed with 5X loading buffer respectively and boiled for 5 min. The proteins were separated on 12.5% SDS-PAGE gel (constant current 14 mA, 90 min). Protein bands were visualized by Coomassie Blue staining.

Protein digestion by trypsin was performed according to the filter-aided sample preparation (FASP) procedure described previously^19^. In brief, 200 μg of proteins for each sample were incorporated into 30 μl SDT buffer (4% SDS, 150 mM Tris-HCl, 100 mM DTT, pH 8.0). The detergent, DTT and other low-molecular-weight components were removed using UA buffer (8 M Urea, 150 mM Tris-HCl, pH 8.0) by repeated ultrafiltration (Microcon units, 10 kDa). Then 100 μl iodoacetamide (100 mM IAA in UA buffer) was added to block reduced cysteine residues and the samples were incubated for 30 min in darkness. The filters were washed with 100 μl UA buffer three times and then 100 μl 25 mM NH_4_HCO_3_ buffer twice. Finally, the protein suspensions were digested with 4 μg trypsin (Promega) in 40 μl 25 mM NH_4_HCO_3_ buffer overnight at 37 °C, and the resulting peptides were collected as a filtrate. The peptides of each sample were desalted on C18 Cartridges (Empore™ SPE Cartridges C18, bed I.D. 7 mm, volume 3 ml, Sigma), dried by vacuum centrifugation and dissolved in 0.1% formic acid in distilled water, then quantified using a NanoDrop 2000 spectrophotometer (Thermo Fisher Scientific) and stored at −80 °C for subsequent LC-MS/MS analysis.

### Liquid chromatography tandem mass spectrometry (LC-MS/MS) analysis

Data Dependent Acquisition (DDA) Mass Spectrometry Assay: All fractions for DDA library generation were analysed by a Thermo Scientific Q Exactive HF mass spectrometer connected to an Easy nLC 1200 chromatography system (Thermo Scientific). The peptide (1.5 μg) was first loaded onto an EASY-SprayTM C18 Trap column (Thermo Scientific, P/N 164946, 2 cm long, 75 µm inner diameter, 3 μm resin), then separated on an EASY-SprayTM C18 LC Analytical Column (Thermo Scientific, ES802, 25 cm long, 75 µm inner diameter, 2 μm resin) with a linear gradient of buffer B (84% acetonitrile and 0.1% formic acid) at a flow rate of 250 nl/min over 90 min. MS detection method was positive ion, the scan range was 300–1,800 m/z, resolution for MS1 scan was 60,000 at 200 m/z, target of AGC (automatic gain control) was 3e6, maximum IT was 25 ms, dynamic exclusion was 30 s. Each full MS–SIM scan followed 20 MS2 scans. The Resolution for MS2 scan was 15,000, AGC target was 5e4, maximum IT was 25 ms, and normalized collision energy was 30 eV.

### Mass spectrometry data processing and statistical analysis

The extracted MS/MS spectra were searched against a protein database combined with *Tropilaelaps mercedesae*, *Apis mellifera*, and all viruses known to infect *Apis mellifera* (37,955 protein sequences in total from NCBI) and appended with the common contaminants using MaxQuant (v. 2.4.2.0). The search parameters were as follows: MS1 match tolerance: 20 ppm for the first search and 6 ppm for the main search; MS2 tolerance: 20 ppm; enzyme: trypsin; allow non-specific cleavage at none end of the peptide; maximum missed cleavages per peptide: 2; fixed modification, Carbamidomethylation; variable modifications: Oxidation and Acetylation (N-term); maximum allowed variable PTM per peptide: 3. A fusion target-decoy approach was used for the estimation of false discovery rate (FDR) and controlled at <1.0% both at peptide and protein levels. Proteins were identified based on at least one unique peptide. Protein quantitation analysis was performed using Perseus (v. 1.6.2.3), and the p-values were Benjamini Hochberg-corrected at 5% FDR.

## Data Records

The LC-MS/MS row data have been deposited in ProteomeXchange Consortium^20^ (http://proteomecentral.proteomexchange.org) via the PRIDE partner repository^21^ with the dataset identifier PXD051347^22^.

## Technical Validation

### Quality evaluation of mass spectrometry data

**Fig. 1:**
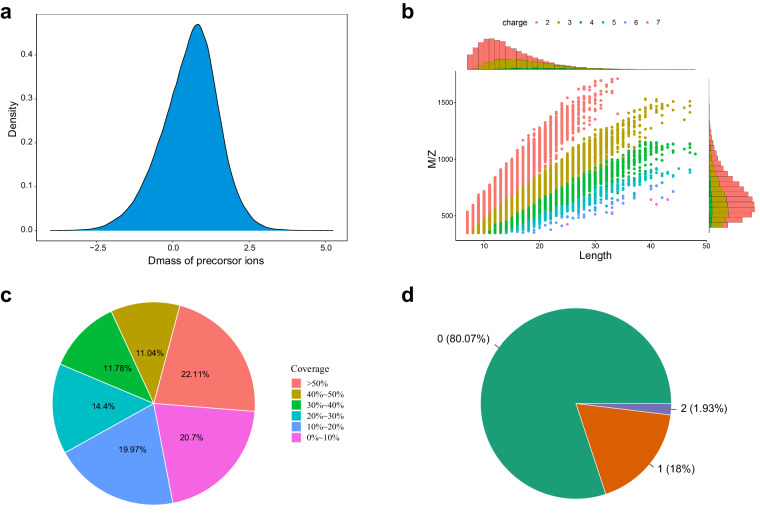
Quality evaluation of mass spectrometry data. (**a**) Distribution of dmass of detected precursor ion. (**b**) Distribution of identified peptide length. (**c**) Distribution of missed cleavage of peptides. (**d**) Distribution of identified protein coverage.

To assess the quality of our mass spectrometry data, we conducted a series of evaluations, including dmass of precursor ion distribution analysis, peptide length distribution analysis, missed cleavage distribution analysis, and protein coverage distribution analysis. The dmass of precursor ions before and after recalibration were normally distributed and mainly distributed within ± 2.5 ppm, showing that the quality accuracy of the mass spectrometry is ideal (Fig. 1a). Most of the peptides detected by mass spectrometry were distributed in the range of 7–20 amino acids, which conform to the general rules of enzymatic digestion and mass spectrometry fragmentation (Fig. 1b). The proportion of peptides with missed cleavages of 0 and 1 was over 98%, confirming that the enzyme digestion is complete and favourable for identification (Fig. 1c). The protein coverage is positively correlated with its abundance in the sample, and in our data, the proportion of proteins with coverage higher than 10% accounts for about 80%, indicating that the protein identification is highly credible (Fig. 1d).

### Reproducibility within the replicates

**Fig. 2:**
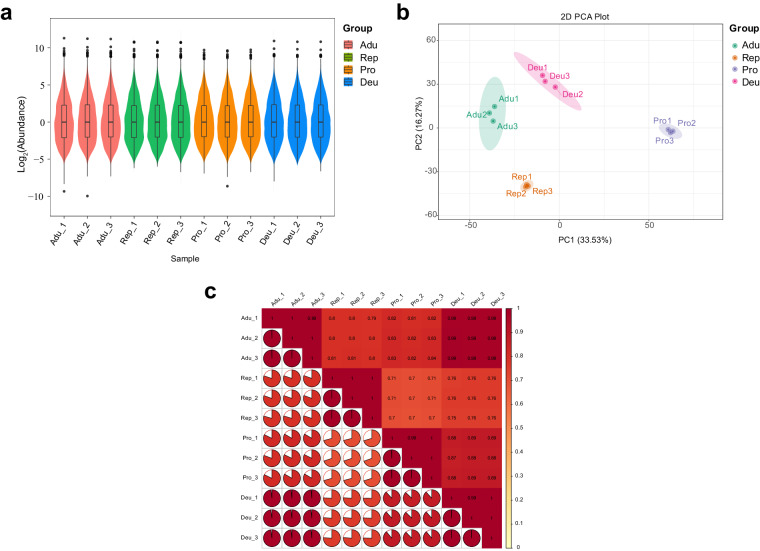
Reproducibility within the replicates. (**a**) Distribution of protein abundance in each sample. The box plots show the degree of dispersion of the protein expression level (abundance), and the violin plots display the distribution and probability density of protein expression levels. (**b**) PCA plot based on the complete proteomic dataset. (**c**) Correlation plot between samples. Pro: protonymphs; Deu: deutonymphs; Adu: adult mites; and Rep: reproductive mites.

To ensure the representativeness of our samples, we collected *T. mercedesae* from 12 honeybee colonies in three apiaries, and prepared three biological replicates for each group of samples. The combination of box plots and violin plots indicates good consistency between our biological replicates (Fig. 2a). Using the unsupervised principal component analysis (PCA) model, the stability and reproducibility of all data sets were monitored. As shown in Fig. 2b, samples from the same group were tightly clustered together and separated from other groups, showing that there is a large overall protein difference between groups, while the variation within the groups is small. The correlation analysis between samples can be used to observe the biological replication between samples within the group. The correlation analysis results showed that the Pearson’s Correlation Coefficient (R) within all four groups of samples is greater than 0.99, which is higher than the R between groups (Fig. 2c), indicating that the obtained differential proteins have high reliability.

## Data Availability

Data analysis procedures have been described in detail in the Methods section. No custom code was used during this study for the curation and/or validation of the dataset.
